# *FCGR2B* and *FCRLB* Gene Polymorphisms Associated with IgA Nephropathy

**DOI:** 10.1371/journal.pone.0061208

**Published:** 2013-04-12

**Authors:** Xu-jie Zhou, Fa-juan Cheng, Yuan-yuan Qi, Yan-feng Zhao, Ping Hou, Li Zhu, Ji-cheng Lv, Hong Zhang

**Affiliations:** Renal Division, Peking University First Hospital, Peking University Institute of Nephrology, Key Laboratory of Renal Disease, Ministry of Health of China, Key Laboratory of Chronic Kidney Disease Prevention and Treatment (Peking University), Ministry of Education, Beijing, People's Republic of China; Institut national de la santé et de la recherche médicale (INSERM), France

## Abstract

**Background:**

IgA nephropathy (IgAN) is a complex syndrome characterized by deposition of IgA and IgA containing immune complexes (ICs) composed of IgG and complement C3 proteins in the mesangial area of glomeruli. The low-affinity receptors for the Fc region of IgG (FcγRs) are involved in autoantibody/immune complex-induced organ injury as well as ICs clearance. The aim of the study was to associate multiple polymorphisms within *FCGR* gene locus with IgAN in a large Chinese cohort.

**Patients and Methods:**

60 single nucleotide polymorphisms (SNPs) spanning a 400 kb range within *FCGR* gene locus were analyzed in 2100 DNA samples from patients with biopsy proven IgAN and healthy age- and sex-matched controls from the same population in Chinese.

**Results:**

Among the 60 SNPs investigated, 15 gene polymorphisms within *FCGR* gene locus (25%) were associated with susceptibility to IgAN. The most significantly associated SNPs within individual genes were *FCGR2B* rs12118043 (p = 8.74*10^−3^, OR 0.76, 95% CI 0.62–0.93), and *FCRLB* rs4657093 (p = 2.28*10^−3^, OR 0.77, 95% CI 0.65–0.91). Both conditional analysis and linkage disequilibrium analysis suggested they were independent signals associated with IgAN. Associations between *FCGR2B* rs12118043 and proteinuria (p = 3.65×10^−2^) as well as gross hematuria (p = 4.53×10^−2^), between FCRLB rs4657093 and levels of serum creatinine (p = 2.67×10^−2^) as well as eGFR (p = 5.41*10^−3^) were also observed. Electronic cis-expression quantative trait loci analysis supported their possible functional significance, with protective genotypes correlating lower gene expressions.

**Conclusion:**

Our data from genetic associations and expression associations revealed potentially pathogenic roles of Fc receptor gene polymorphisms in IgAN.

## Introduction

IgA nephropathy (IgAN) was described histologically for the first time in 1968 by Berger and Hinglais as *les dépôts intercapillaires d*'*IgA-IgG* (intercapillary deposits of IgA-IgG)[1]. It was characterized by the deposition of IgA in the mesangial area of glomeruli, and proliferation of the glomerular mesangium with deposition of immune complexes composed of IgG and complement C3 proteins. Current data indicates that at least four hits contribute to development of IgA nephropathy: aberrant glycosylation of IgA1, synthesis of antibodies directed against galactose-deficient IgA1, binding of the galactose-deficient IgA1 by the anti-glycan/glycopeptides antibodies to form immune complexes (ICs), and accumulation of these complexes in the glomerular mesangium to initiate renal injury[2]. But the presence of circulating IgA1-containing ICs is not unique to patients with IgAN. IgA1-IgG ICs can also be detected in persons without apparent renal disease[3], [4], [5]. Anyhow, the pathogenic importance of ICs has been widely recognized, with plenty of evidence such as, serum levels of IgG antibodies specific for galactose deficient IgA1 correlated with disease severity[3]; the pathogenic circulating IgA1-IgG ICs in patients with IgAN are relatively large (>800 kD)[6]; ICs from patients with IgAN containing galactose-deficient IgA1 bind to the cells more efficiently than do uncomplexed IgA1 or ICs from healthy controls[7]; complexes with galactose- deficient IgA1 induce cultured human mesangial cells to proliferate, secrete extracellular matrix components, release cytokines and further interfere mesangial cell-podocyte crosstalk[8]. However, factors influencing the formation/composition of these ICs and the intrinsic mechanisms leading to cell activation and glomerular damage were still not clearly elucidated.

Molecular mechanisms of autoantibody/immune complex-induced organ injury as well as ICs clearance often involve two main components, namely the low-affinity receptors for the Fc region of IgG (FcγRs) and the complement system[9], [10]. Several lines of functional evidences were emerging to the important role of complement in IgAN, especially alternative pathway[11], [12], [13], [14]. Recent genome association studies (GWAS) also indicated that genetic variants of CFH/CFHR were associated with IgAN, further highlighting the inherited and pathogenic role of complement pathway in IgAN[15]. Fcγ Rs contribute to the regulation of a multitude of immune and inflammatory responses. Polymorphisms in the genes encoding FcγRs (*FCGR*) have been associated with susceptibility to a number of autoimmune or inflammatory diseases. However, few studies have been conducted to address the functional role of FcγRs as well as *FCGR* gene polymorphisms in IgAN. Up to date, only two genetic studies involving less than 200 patients with IgAN had been reported to associate reported *FCGR* gene variants with IgAN, but with contradictory conclusions [16], [17]. So in the current study, we aimed to associate multiple polymorphisms within *FCGR* gene locus with IgAN in a large Chinese cohort comprised by more than 2000 samples to further determine its genetic role in IgAN.

## Materials and Methods

### Patients and Controls

The study population consisted of 1,200 IgAN cases and 900 healthy controls of Chinese Han ancestry from north of China. All of the patients with IgAN were confirmed by renal biopsy. Patients with secondary IgAN or with other comorbid renal diseases were excluded. Clinical and laboratory data at the time of diagnosis were collected for each patient. Written informed consent was obtained from each patient. This study complied with the Declaration of Helsinki and was approved by the medical ethics committee of Peking University First Hospital.

### DNA extraction

Genomic DNA was isolated from whole peripheral blood using a modified salt extraction technique. DNA concentration and quality [optical density (OD) 260/OD 280 and OD 260/OD 230 measurements] were determined by a Nanodrop ND1000 spectrophotometer (Thermo Fisher Scientific, Waltham, MA, USA).

### Genotyping

**Figure 1:**
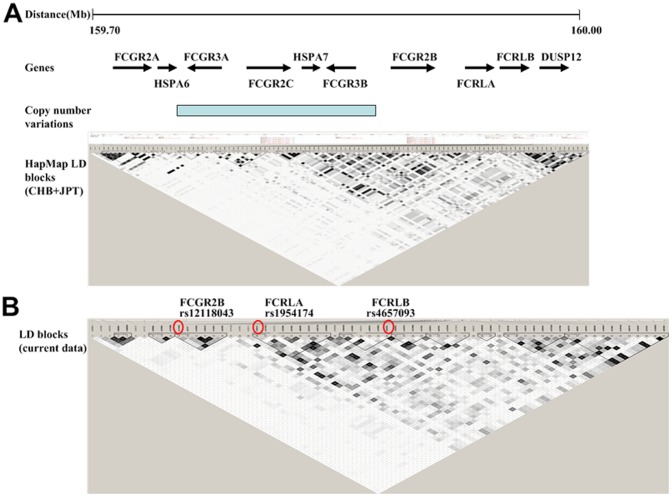
Lack of extensive haplotype blocks with FCGR gene loci in 1q23. (**Figure 1A**) The linkage disequilibrium (LD) blocks were derived from the HapMap3 Asian CHB and JPT dataset (http://www.hapmap.org). CHB: 80 Han Chinese from Beijing, China; JPT: 82 Japanese in Tokyo, Japan. The locus was complicated by multiple kinds of genetic variations including single-nucleotide polymorphisms (SNPs) as well as copy number variations with low linkage disequilibrium. (**Figure 1B**) The linkage disequilibrium (LD) blocks of SNPs in the current study. The SNPs associated with IgAN were marked by circle.

Genes encoding low-affinity FcγRs including *FCGR2A*, *FCGR3A*, *FCGR2C*, *FCGR3B* and *FCGR2B* locate in 1q23 spanning a 200-Kb region (**Figure 1A**). As genes within the locus were of low but complex linkage disequilibrium (LD), an extended region with 100 kb upstream and 100 kb downstream of the FCGR gene locus was analyzed. SNPs were genotyped on a customized Illumina Human 610-Quad BeadChip platform as previously reported[18]. The genotyping call rate was 99.9%. A total of 60 SNPs were analyzed in the current association study.

### Cis-eQTL (cis-expression quantative trait loci) analysis in HapMap samples

Normalized mRNA data from Epstein–Barr virus (EBV)-transformed lymphoblastoid cell lines of HapMap3 CHB (80 Han Chinese from Beijing, China) and JPT (82 Japanese in Tokyo, Japan) population were obtained from the database of the Gene Expression Variation (GENEVAR) project at the Wellcome Trust Sanger Institute (http://www.sanger.ac.uk/humgen/genevar/)[19].

### Statistical analysis

The SNPs meeting the quality control criteria of less than 1% overall missing data, consistency with Hardy-Weinberg Equilibrium genotype frequency expectations (P>0.05) were included. Association analysis was performed using the trend test in PLINK controlling population stratification. Odd ratio (OR) values were presented for the minor allele of a SNP. Linkage disequilibrium (LD) was tested using Haploview (version 4.2, http://www.broad.mit.edu/mpg/haploview) as well as the EM algorithm within PLINK. The association between SNPs and CNV was determined by chi-square test and Person's correlation coefficients were calculated for LD estimation[20], [21]. Power was calculated by Power and Sample Size Calculations Software (version 3.0, http://biostat.mc.vanderbilt.edu/PowerSampleSize). Spearman's coefficient were calculated to correlate genotypes and gene expressions in cis-eQTL analysis as reported[19].

## Results

### Fc receptor gene polymorphisms associated with susceptibility to IgAN

As can be seen from **Table 1**, among the 60 SNPs investigated, 15 SNPs within *FCGR* gene locus (25%) were associated with susceptibility to IgAN, suggesting likely true associations. Among SNPs within the classical FCGR genes, rs12118043A showed statistically significant disease association (p = 8.74*10^−3^, OR 0.76, 95% CI 0.62–0.93). A SNP of rs4657093C (p = 2.28*10^−3^, OR 0.77, 95% CI 0.65–0.91) within *FCRLB* showed the most significant association signal in the extended region (**Table S1**). Power calculations indicated that we had at least 99% power to detect loci with allelic frequencies >0.10 and relative risk >1.5 assuming an α-level of 0.05 (P<0.05) in the current study.

**Table 1:** Associations between FCGR gene polymorphisms and susceptibility to IgAN.

SNP	Bp	Gene	Function class	Minor Allele	MAF case/control (%)	Trend test p values	Allele OR (95% CI)
rs4657039	159729352	intergenic		G	24.37/27.77	1.28*10^−2^	0.84 (0.73–0.96)
rs12118043	159913448	*FCGR2B*	intron	A	8.42/10.81	8.74*10^−3^	0.76 (0.62–0.93)
rs1954173	159944408	*FCRLA*	intron	G	16.00/18.42	3.86*10^−2^	0.84 (0.72–0.99)
rs1954174	159944431	*FCRLA*	intron	T	42.20/38.69	2.19*10^−2^	1.16 (1.02–1.31)
rs2333749	159951245	*FCRLA*	nearGene-3′	T	22.36/25.22	3.08*10^−2^	0.85 (0.74–0.97)
rs10917750	159952215	intergenic		C	15.62/18.85	5.89*10^−3^	0.80 (0.68–0.94)
rs10494356	159953885	intergenic		G	42.67/39.30	2.82*10^−2^	1.15 (1.02–1.30)
rs7549830	159955055	intergenic		C	50.50/46.90	2.07*10^−2^	1.16 (1.02–1.31)
rs1891019	159958057	*FCRLB*	nearGene-5′	T	47.61/51.72	8.46*10^−3^	0.85 (0.75–0.96)
rs4657093	159959627	*FCRLB*	intron	C	13.91/17.35	2.28*10^−3^	0.77 (0.65–0.91)
rs1891020	159961078	*FCRLB*	intron	A	15.95/18.81	1.52*10^−2^	0.82 (0.70–0.96)
rs12079477	159961481	*FCRLB*	intron	G	48.11/51.72	2.09*10^−2^	0.87 (0.77–0.98)
rs1417582	159962712	*FCRLB*	intron	T	16.01/18.92	1.38*10^−2^	0.82 (0.70–0.96)
rs1503813	159967303	intergenic		G	18.30/21.01	2.83*10^−2^	0.84 (0.72–0.98)
rs905594	160007898	*ATF6*	intron	C	37.43/34.48	4.93*10^−2^	1.14 (1.00–1.29)

### Linkage disequilibrium and conditional analysis suggested independent associations

The LD statistics based on haplotype frequencies estimated via the EM algorithm within PLINK showed the most significantly associated SNPs rs12118043, rs1954174, and rs4657093 within *FCGR2B*, *FCRLA*, *FCRLB* were in low LD (**Figure 1B**). rs12118043 has an r^2^ = 0.016 with rs1954174, rs12118043 has an r^2^ = 0.001with rs4657093, and rs1954174 has an r^2^ = 0.123 with rs4657093. rs12118043 (p = 5.00*10^−3^) remained significantly associated with IgAN after conditional logistic regression incorporating rs4657093, suggesting they were independently associated with IgAN. However, the association with rs1954174 became non-significant in conditional analysis (p = 0.17).

### Association analysis between *FCGR3B* copy numbers and *FCGR2B* rs12118043 genotypes in HapMap samples indicated weak linkage disequilibrium

Fc receptor genes likely arose by segmental duplications during evolution and it was reported that copy number variations in 1q23 involved *FCGR3A*, *FCGR2C* and *FCGR3B* [20], [22], [23], [24], [25]. To determine whether the effect of *FCGR2B* rs12118043A in risk of IgAN originated from its independent contribution or was in LD with CNVs at this locus, we derived data of *FCGR3B* copy numbers and *FCGR2B* rs12118043 genotypes from HapMap samples. As it was widely accepted that it was difficult to genotype FCGR3B copy numbers accurately by a single method, the *FCGR3B* copy numbers we applied were from an integrated suite of five assays[21], which provided the bases of reliability and precision for further analysis. As can be seen from **Table 2**, *FCGR2B* rs12118043 risk genotypes associated with lower *FCGR3B* copy numbers in Caucasians (p = 0.03) but not in people from Asia or Africa. When individual genotypes were coded as 1, 2, and 3, represented homozygote AA, heterozygote AC, and homozygote CC, respectively. The square of Pearson's correlation coefficient (r^2^) calculated for copy number of *FCGR3B* and *FCGR2B* rs12118043 genotypes were 0.01 (p = 0.35) in HapMap Asians and 0.07 (p = 0.02) in Caucasians respectively. The data indicated that *FCGR2B* rs12118043A was in weak LD with FCGR3B low copy numbers, especially in peoples of Asian and African ancestry, which was also consistent to previous reports including several other different SNPs[20].

**Table 2:** Associations between *FCGR3B* copy numbers and *FCGR2B* rs12118043 genotypes in HapMap samples.

	*FCGR2B* rs12118043 Genotype					
*FCGR3*B CN	CHB+JPT (n = 87)		CEU (n = 80)		YRI (n = 78)	
	AA+AC	CC	AA+ AC	CC	AA+ AC	CC
0	––	––	1(3.1)	––	––	––
1	1(8.3)	8(10.7)	3(9.4)	1(2.0)	––	11(14.1)
2	7(58.3)	44(58.7)	26(81.2)	34(69.4)	––	59(75.6)
3	3(25.0)	23(30.7)	2(6.2)	12(24.5)	––	6(7.7)
4	1(8.3)	––	––	2(4.1)	––	2(2.6)
P values	p = 0.14		p = 0.03			

As samples of rs12118043 genotype AA were few, dominant model with AA+AC versus CC was applied. Likelihood ratio test was applied as more than 2 cells had expected count less than 5 in every Chi-square test.

### Fc receptor gene polymorphisms associated with severity of IgAN

The above data indicated IgAN associated SNPs *FCGR2B* rs12118043 and *FCRLB* rs4657093 may impact IgAN susceptibility independently. We further checked their correlations with severity of IgAN accordingly. The parameters included blood pressure, serum creatinine, urine protein, uric acid, estimated glomerular filtration rate (eGFR) calculated based on MDRD formula modified for Chinese population[26], and Hass pathology grade classification[27]. It was indicated that *FCGR2B* rs12118043 associated with proteinuria, with protective genotypes correlating lower levels of proteinuria (AA+AC vs. CC 1.54±1.13 g/day vs. 2.00±1.99g/day, p = 3.65×10^−2^) and lower frequency of gross hematuria (26.6% vs. 34.0%, p = 4.53×10^−2^). *FCRLB* rs4657093 protective genotypes associated with smaller age of onset (31.31±10.91 vs. 32.96±11.28, p = 2.58×10^−2^), and better renal function including higher levels of eGFR (CC+CT vs. TT 91.62±26.74 ml/min*1.73 m^2^ vs. 80.07±27.41 ml/min*1.73 m^2^, p = 5.41*10^−3^) and lower levels of serum creatinine (92.56±36.51 µmol/L vs. 107.67±67.79 µmol/L, p = 2.67×10^−2^) (**Table 3**).

**Table 3:** Associations between Fc receptor gene polymorphisms and severity of IgAN.

Clinical Parameters	*FCGR2B* rs12118043			*FCRLB* rs4657093		
	AA+AC	CC	*p*	CC+CT	TT	*p*
Age (year)	33.27±11.29	32.41±11.20	0.33	31.31±10.91	32.96±11.28	**2.58×10^−2^**
Gender (Male %)	53.9	54.8	0.82	46.6	45.0	0.63
SBP (mmHg)	125±17	124±18	0.61	123±18	124±17	0.14
DBP (mmHg)	78±12	79±13	0.84	78±13	79±13	0.25
eGFR(ml/min/1.73 m^2^)	84.35±22.96	82.05±28.44	0.62	91.62±26.74	80.07±27.41	5.41*10^−3^
Scr (umol/L)	93.94±106.62	106.62±67.31	0.23	92.56±36.51	107.67±67.79	**2.67×10^−2^**
24 hour UTP (g/day)	1.54±1.13	2.00±1.99	**3.65×10^−2^**	1.73±1.38	1.98±1.99	0.38
Gross Hematuria (%)	26.6	34.0	**4.53×10^−2^**	36.9	31.4	0.08
Uric Acid (umol/L)	351.55±104.34	347.84±112.17	0.68	343.53±106.79	350.08±112.38	0.39
LDL-c (mmol/L)	2.87±1.22	3.14±5.21	0.48	3.39±7.86	3.00±3.12	0.24
Serum IgA (g/L)	2.86±1.01	2.83±1.08	0.89	2.81±0.95	2.85±1.10	0.82
HASS Grade (%)			0.77			0.55
I	39.7	38.0		37.3	38.7	
II	9.6	6.1		9.7	5.4	
III	37.0	38.5		37.3	38.7	
IV	5.5	8.4		8.2	7.7	
V	8.2	8.9		7.5	9.4	

SBP: systolic blood pressure; DBP: diastolic blood pressure; eGFR: estimated glomerular filtration rate (eGFR) calculated based on MDRD formula modified for Chinese population; Scr: serum creatinine; 24 hour UTP: 24 hours total urine protein; LDL-c: low density lipoprotein cholesterol

### Cis-eQTL (cis-expression quantative trait loci) analysis supported possible functional significance of Fc receptor gene polymorphisms

**Figure 2:**
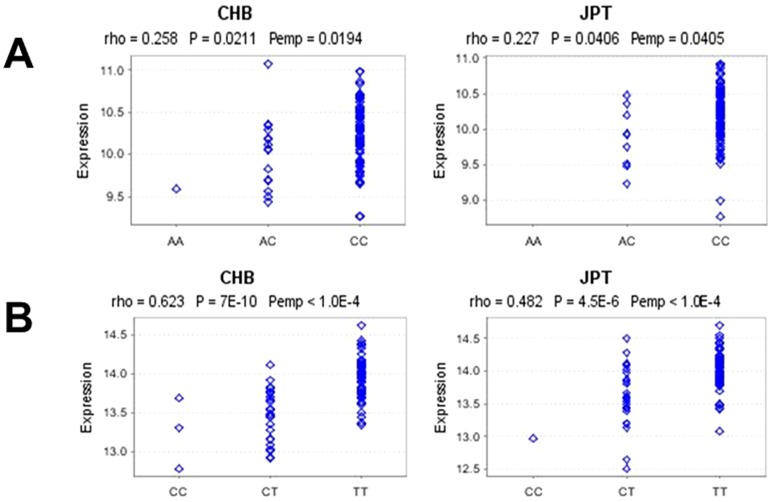
Cis-eQTL (cis-expression quantative trait loci) analysis of Fc receptor gene polymorphisms. (**Figure 2A**) associations between genotypes of *FCGR2B* rs12118043 and *FCGR2B* expression. (**Figure 2B**) associations between genotypes of FCRLB rs4657093 and FCRLA expression. eQTLs have been studied in lymphoblastoid cell lines (LCLs) from the HapMap3 Asian populations. The distance from the genomic location of the transcription start site (TSS) to SNP genomic location was less than 1 Mb. Spearman's rank correlation coefficients (rho) and p values were presented. The data was derived from Genevar project (http://www.sanger.ac.uk/resources/software/genevar). CHB: 80 Han Chinese from Beijing, China; JPT: 82 Japanese in Tokyo, Japan.

A common hypothesis held that common SNPs impact disease by altering abundance of gene transcripts. We thus checked correlations between genotypes and gene expressions in cis (cis eQTL or local eQTL maps to the location whose expression levels are associated with genetic variation located physically close to the gene)[19]. By analyzing correlations between genotypes and nearby gene expression within a 1 Mb distance, we observed significant associations between genotypes of *FCGR2B* rs12118043 and *FCGR2B* expression (**Figure 2A**), and between genotypes of *FCRLB* rs4657093 and *FCRLA* expression (**Figure 2B**) in HapMap3 Asians (p<0.05 both in CHB and JPT populations), in which the protective alleles always associated with lower gene expressions. The data further supported *FCGR2B* rs12118043 and *FCRLB* rs4657093 were likely true disease associated SNPs.

## Discussion

FcγRs are now recognized as the dominant molecules responsible for coupling the recognition of antigens by IgG antibodies to the cellular effector pathways of macrophages, neutrophils, natural killer cells and mast cells[28]. They are a critically involved in the maintenance of peripheral tolerance, regulating dendritic cell maturation and plasma cell survival[29]. Gene polymorphisms of FCGR gene family have been associated with multiple immune-related diseases including human autoimmune, infectious or malignant diseases. Novel therapeutic strategies targeting FcγRs especially on FCGR gene polymorphisms are emerging [28], [30], [31], [32]. Fc receptor-like (FcRL) proteins are a family of cellular receptors homologous to FcγRs that are preferentially expressed on B lineage cells. The extracellular ligands as well as functions of these receptors are still unknown or controversial [33], [34], [35], [36]. However, several genetic studies also revealed FCRL gene variants were associated with multiple immune-related diseases, no matter in genome-wide association studies or in candidate gene based studies [37], [38], [39], [40], [41]. All the above highlighted the central bridging roles of FcγRs and FcRLs in immunity with their genetic variants to make up a sort of susceptibility background.

IgAN is a complex syndrome characterized by deposition of IgA and IgA containing immune complexes composed of IgG and complement C3 proteins in the mesangial area of glomeruli. As FcγRs and FcRLs were expressed in B cells and mesangial cells and they were the effector molecules in mediating antibody/immune complex effects, the roles of FcγRs and FcRLs in IgAN will be of particular interest. In the present study, we investigated 60 SNPs in 1q23 spanning an extended region with 100 kb upstream and 100 kb downstream of the FCGR gene locus. We observed that 15 gene polymorphisms within *FCGR* gene locus (25%) were associated with susceptibility to IgAN. The most significantly associated SNPs were rs12118043, rs1954174, and rs4657093 within *FCGR2B*, *FCRLA*, and *FCRLB* respectively. Both conditional analysis and linkage disequilibrium analysis suggested they were independent signals associated with IgAN. Interestingly, LD between *FCGR2B* rs12118043 and *FCGR3B* copy numbers was higher in Caucasians than that in Asians and Africans, supporting complex genetic architectures in the gene locus. Integration of different genetic variants in different populations will reveal more information about disease pathogenesis. Future studies involving copy number variations in the gene locus will be deserved, especially in Asians and Africans. In addition, we observed associations between *FCGR2B* rs12118043 and proteinuria as well as gross hematuria, between *FCRLB* rs4657093 and levels of serum creatinine as well as eGFR, which indicated that the variants not only impacted disease susceptibility but also disease severity. At last, cis-eQTL analysis supported their possible functional significance, with protective genotypes correlating lower gene expressions. As that was observed in infectious diseases [31], [42], *FCGR2B* protective genotypes correlated lower gene expression and lower frequency of gross hematuria, latter of which was significantly associated mucosal infection and was an important indicator of episodes of IgAN. Receptor deficiency may down-regulate responses mediated by the receptor. For example, as FcγR2b was the only one inhibitory receptor to suppress downstream events such as cellular proliferation, phagocytosis, and inflammatory cytokine release, *FCGR2B* rs12118043 protective genotypes may have better humoral immunity, i.e. produce less autoantibodies and mediate balanced inflammation[31]. The data above from two different sorts of associations – genetic association and expression association, suggested the associations were likely true. Anyhow, the association in IgAN was relatively weak and more detailed pathogenesis was still not clear. Further assays in gene-target animal models or cell lines will be welcomed, which will explain more about the specific role of FcγRs in IgAN.

In conclusion, we investigated multiple SNPs spanning a 400 kb range within FCGR gene locus in 2100 Chinese, and observed significant associations between *FCGR2B*, *FCRLA*, and *FCRLB* gene variants and IgAN. Both conditional analysis and linkage disequilibrium analysis suggested they were independent signals associated with IgAN. Genetic associations and expression associations suggested the Fc receptor gene polymorphisms play potentially pathogenic roles in IgAN. Future studies involving more genetic variants in diverse populations and investigations of specific pathogenesis will be deserved.

## Supporting Information

Table S1 **Genotype counts of investigated SNPs in IgAN patients and controls.**
(DOC)
