# Supplementary material for: FCGR2B and FCRLB Gene Polymorphisms Associated with IgA Nephropathy
Source: PLoS One. 2013 Apr 12;8(4):e61208. doi: 10.1371/journal.pone.0061208 (PMC3625155; doi:10.1371/journal.pone.0061208)
Supplement: Table S1 — Genotype counts of investigated SNPs in IgAN patients and controls. (DOC) [file pone.0061208.s001.doc]

**Table S1**: Genotype counts of investigated SNPs in IgAN patients and controls

| SNP | Allele1 | Allele2 | Case | Control | p |
| --- | --- | --- | --- | --- | --- |
| rs3922744 | C | T | 196/616/381 | 139/451/310 | 0.4672 |
| rs11265601 | C | T | 0/60/1133 | 0/31/869 | NA |
| rs6698806 | G | T | 53/461/680 | 55/329/515 | 0.1861 |
| rs2165090 | T | G | 221/571/402 | 158/476/266 | 0.0604 |
| rs4657039 | G | A | 74/434/686 | 61/378/461 | **0.01714** |
| rs6696854 | C | T | 97/466/631 | 80/385/434 | 0.1171 |
| rs10800309 | G | A | 90/447/657 | 70/375/455 | 0.116 |
| rs1801274 | C | T | 120/495/579 | 97/405/398 | 0.1521 |
| rs511278 | T | C | 0/137/1054 | 0/113/787 | NA |
| rs2099684 | G | A | 89/489/616 | 63/344/491 | 0.3753 |
| rs7539036 | A | G | 0/194/994 | 0/118/777 | NA |
| rs12128686 | C | T | 1/197/993 | 0/125/772 | 0.1788 |
| **rs12118043** | A | C | 8/185/1001 | 4/187/709 | **0.006321** |
| rs723177 | T | C | 64/436/694 | 59/338/503 | 0.3971 |
| rs1256286 | T | C | 268/569/357 | 199/461/239 | 0.1855 |
| rs1832739 | T | G | 268/568/356 | 198/462/239 | 0.1789 |
| rs1340976 | C | A | 258/567/369 | 187/462/251 | 0.1933 |
| rs4657090 | A | G | 63/437/694 | 59/337/504 | 0.3781 |
| rs17413333 | A | G | 148/572/474 | 133/440/327 | 0.1501 |
| rs1538972 | G | A | 0/42/1152 | 0/22/878 | NA |
| rs1954173 | G | A | 31/320/843 | 27/278/594 | 0.08673 |
| rs1954174 | T | C | 215/577/401 | 129/438/333 | 0.05145 |
| rs12135523 | G | A | 58/401/735 | 45/323/532 | 0.5176 |
| rs7523762 | T | C | 0/44/1150 | 0/24/876 | NA |
| rs1934908 | C | T | 75/433/686 | 61/340/499 | 0.6443 |
| rs2275603 | A | G | 214/587/393 | 173/426/301 | 0.6482 |
| rs6683580 | A | G | 135/549/510 | 110/396/393 | 0.6309 |
| rs2333749 | T | G | 67/400/727 | 61/332/507 | 0.09859 |
| rs10917750 | C | T | 32/309/853 | 31/278/591 | **0.01767** |
| rs12042893 | T | C | 135/552/506 | 110/401/389 | 0.6807 |
| rs16856590 | C | T | 215/607/372 | 178/425/297 | 0.251 |
| rs10494356 | G | A | 217/585/392 | 136/435/329 | 0.08332 |
| rs7549830 | C | T | 297/612/285 | 183/477/240 | **0.03842** |
| rs1891018 | C | A | 0/45/1149 | 0/25/875 | NA |
| rs1891019 | T | C | 262/612/319 | 225/482/193 | **0.01489** |
| rs11590932 | C | T | 4/133/1057 | 3/103/793 | 0.9744 |
| **rs4657093** | C | T | 23/286/884 | 27/259/614 | **0.008689** |
| rs1891020 | A | G | 32/317/845 | 31/277/591 | **0.04548** |
| rs12079477 | G | A | 279/590/324 | 242/447/211 | 0.07062 |
| rs12728899 | A | G | 10/210/974 | 13/136/750 | 0.1481 |
| rs1417582 | T | C | 32/317/841 | 31/277/586 | **0.04152** |
| rs1503813 | G | A | 38/361/795 | 35/309/556 | 0.07188 |
| rs6427623 | T | C | 70/439/682 | 47/348/505 | 0.6195 |
| rs11576107 | C | T | 168/559/466 | 129/423/348 | 0.9775 |
| rs12749327 | T | C | 1/116/1077 | 2/63/835 | 0.06438 |
| rs905589 | T | C | 41/367/785 | 35/312/553 | 0.1211 |
| rs6699725 | T | C | 121/538/535 | 81/419/400 | 0.6216 |
| rs16859935 | A | C | 8/189/997 | 4/133/763 | 0.63 |
| rs10799941 | G | T | 238/599/357 | 159/441/299 | 0.1883 |
| rs17415059 | T | C | 4/166/1024 | 5/122/773 | 0.731 |
| rs6657266 | T | C | 52/387/755 | 42/306/552 | 0.6708 |
| rs1027702 | C | T | 128/515/550 | 97/421/382 | 0.2191 |
| rs1063178 | T | C | 266/588/340 | 164/463/273 | 0.07391 |
| rs1063179 | T | C | 52/387/755 | 42/307/551 | 0.6406 |
| rs3820449 | T | C | 130/512/551 | 84/367/444 | 0.2416 |
| rs12745240 | A | G | 266/591/337 | 164/465/271 | 0.07358 |
| rs1417580 | C | A | 128/518/548 | 97/422/380 | 0.2266 |
| rs4657101 | C | A | 113/505/576 | 86/404/410 | 0.4525 |
| rs905594 | C | T | 174/545/474 | 106/409/385 | 0.1232 |
| rs2070151 | T | C | 113/499/582 | 82/407/410 | 0.2774 |
